# 

**Published:** 1893-09

**Authors:** 


### CONTENTS.

### *EDITORIALS.*

PAGE

Neural Analysis, . . . . . . . . . . . . 449
Dr. Wells on Intermittent Fever, . . . . . . . . 450

### *MISCELLANEOUS.*

Potentiation Physiologically Proven, . . . . . . 450
Appendicitis. Jas. Lilienthal, M. D., . . . . . . 458
Complexion and Constitution, . . . . . . . . . 462
The Suppression of Symptoms, . . . . . . . . 467
Provings and Clinical Observations with High Potencies. Malcolm Macfarlan, M. D., . . . . . . . . . 468
Gleanings. F. H. Lutze, M. D., . . . . . . . . 474

### *BOOK NOTICES.*

Psychopathia Sexualis. By Dr. R. Von Krafft-Ebing, . . . . 478
Transactions of the Forty-fifth Session of The American Institute of Homœopathy. By Pemberton Dudley, M. D., . . . . 480
A Chapter on Cholera for Lay Readers. By Walter Vought, M. D., 480

